# Case Report: Hypothalamic demyelinating lesion preceding lymphoma arising outside the central nervous system

**DOI:** 10.3389/fonc.2025.1553142

**Published:** 2025-09-22

**Authors:** Narushi Sugii, Hiroyoshi Kino, Tetsu Suzuki, Noriaki Sakamoto, Makoto Shibuya, Ken Akimoto, Masahide Matsuda, Eiichi Ishikawa

**Affiliations:** ^1^ Department of Neurosurgery, Institute of Medicine, University of Tsukuba, Tsukuba, Japan; ^2^ Department of Neurology, Institute of Medicine, University of Tsukuba, Tsukuba, Japan; ^3^ Department of Diagnostic Pathology, Institute of Medicine, University of Tsukuba, Tsukuba, Japan; ^4^ Central Clinical Laboratory, Hachioji Medical Center, Tokyo Medical University, Hachioji, Japan

**Keywords:** case report, demyelinating lesion, diffuse large B-cell lymphoma, outside the central nervous system, sentinel lesion

## Abstract

Nonneoplastic demyelinating brain lesions, known as sentinel lesions, occasionally precede primary central nervous system lymphomas (PCNSL). However, cerebral sentinel lesions associated with lymphoma “outside” the central nervous system (CNS) have not been reported. Here, we describe the case of a 76-year-old female who presented with rapidly progressive visual field disturbances. Magnetic resonance imaging revealed a hypothalamic lesion, and a biopsy demonstrated inflammatory demyelination with T-lymphocyte and macrophage infiltration, but no evidence of lymphoma. Steroid therapy was initiated only after biopsy. The lesions responded well to steroids and almost disappeared, and the patient was discharged with improvement in all symptoms except for diabetes insipidus. Three months later, the patient developed systemic symptoms, including osteolytic bone and skull lesions and multiple organ failure. A second biopsy of a skull lesion revealed diffuse large B-cell lymphoma (DLBCL), a non-germinal center B-cell-like subtype. Despite initiating chemotherapy, the patient died five months after the initial biopsy, although no recurrence of the brain lesion was observed. This case is the first to document a brain sentinel lesion preceding systemic DLBCL without associated cerebral lymphoma. It highlights the similarities with previously reported sentinel lesions in PCNSL, such as inflammatory demyelination and steroid responsiveness, while raising questions regarding the underlying mechanisms and challenges of early diagnosis. We emphasize the importance of considering sentinel lesions in patients with tumefactive inflammatory demyelination characterized by T-lymphocyte-dominant infiltration.

## 1 Introduction

Primary central nervous system lymphoma (PCNSL) is a relatively rare type of non-Hodgkin lymphoma, accounting for approximately 2% of newly diagnosed brain tumors (1). Approximately 90% of PCNSL cases are diffuse large B-cell lymphoma (DLBCL), and it is typically diagnosed by biopsy of brain tissue (2).

PCNSL is sometimes preceded by demyelinating lesions in the brain, called “sentinel lesions” (3). Although the exact mechanism of sentinel lesions is unknown, they are nonneoplastic, and their biopsies do not yield a diagnosis of lymphoma. While approximately 20 cases of sentinel lesions associated with PCNSL have been reported (3–17), there have been no reports of sentinel lesions of the brain associated with DLBCL arising outside the central nervous system (CNS).

Here, we report the first documented case of a hypothalamic sentinel lesion preceding systemic DLBCL without CNS involvement, where the first biopsy did not lead to a diagnosis, but the second biopsy confirmed the pathology.

## 2 Case description

**Figure 1:**
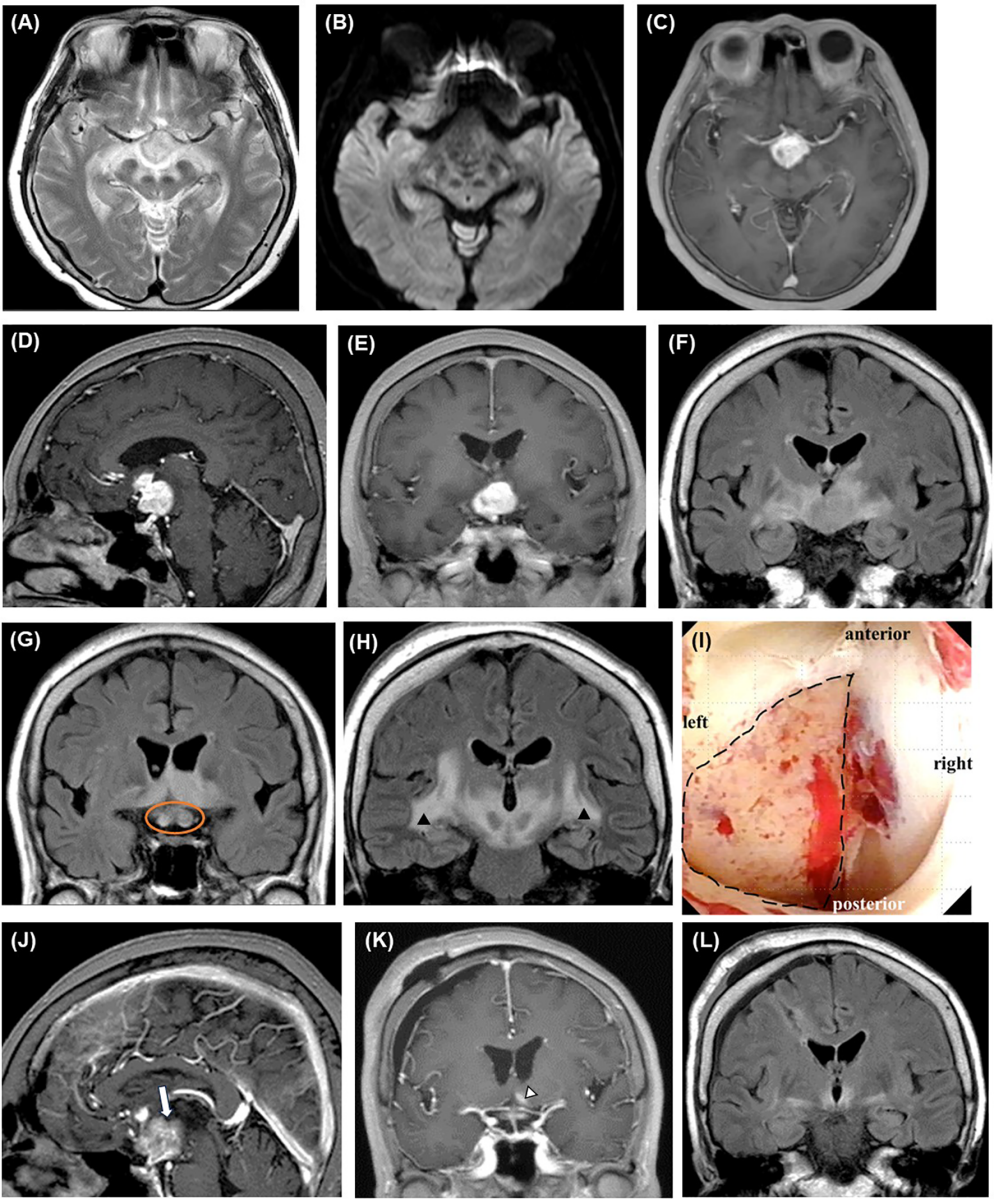
Perioperative imaging findings of initial biopsy. Preoperative brain MRI reveals a well-enhanced mass lesion primarily located in the pituitary stalk and left hypothalamus on axial **(C)**, sagittal **(D)**, and coronal **(E)** T1WIs with contrast medium. The lesion shows high signal intensity on T2WI **(A)** and FLAIR (**(F)**, coronal section) without diffusion restriction **(B)**. The optic nerves are swollen (orange circle) **(G)**, and high-intensity lesions extensively spread along the white matter tracts on the FLAIR coronal sections (arrowheads) **(H)**. Intraoperative neuroendoscopic imaging of the third ventricle **(I)** shows the lesion after the removal of the ependymal layer (area in the dotted circle). Contrast-enhanced MRI performed on the day after surgery shows a contrast defect (blank arrow), indicating that the specimen has been successfully collected from the lesion [**(J)**, sagittal view]. Two weeks after the start of steroid therapy, only a trace of contrast-enhanced lesion (blank arrowhead) (**(K)**, contrast-enhanced T1WI coronal view) with small surrounding FLAIR high-signal area is observed [**(L)**, FLAIR coronal view]. FLAIR, fluid-attenuated inversion recovery; MRI, magnetic resonance imaging; WI, weighted image.

A 76-year-old female with no significant medical history except for hypertension and dyslipidemia was transferred to our hospital with rapidly progressive visual field disturbances and impaired consciousness for over two weeks. The patient had no signs of papilloedema; however, her left eye was nearly blind. Head magnetic resonance imaging (MRI) revealed a slightly low-signal intensity mass lesion on both T1- and T2-weighted images (WI) from the optic chiasm to the hypothalamus, which was markedly enhanced by Gd-based contrast medium (**Figures 1A–H**). The lesion did not exhibit diffusion restrictions and was accompanied by an extensive Fluid-Attenuated Inversion Recovery (FLAIR) high-signal region at the periphery. A contrast-enhanced whole-body computed tomography scan showed no significant findings, such as mass or bone lesions. Anterior pituitary function, evaluated by a morning fasting blood test, was intact, and the serum soluble interleukin-2 receptor (sIL-2R) level was 193 ng/mL (negative). Although narrowing down the diagnosis preoperatively was challenging, we suspected PCNSL and decided to perform an endoscopic biopsy using a flexible scope targeting the left hypothalamic lesion compressing the third ventricle. No steroids were administered preoperatively.

An ependymal layer covered the lesion, and the third ventricle was stenotic because of compression from the lesion. After removing the ependium, we collected more than ten samples from the left hypothalamus. The lesion appeared white and did not bleed significantly (**Figure 1I**). The enhancement defect (i.e., biopsy site) was confirmed on MRI after surgery (**Figure 1J**). We started 10 mg/day of dexamethasone immediately after the biopsy, and the response to steroids was excellent, with rapid improvement in consciousness and imaging findings (**Figures 1K, L**). However, the patient developed diabetes insipidus, likely due to the operative stress on the hypothalamus. She was discharged home on a maintenance dose of 2.5 mg/day of dexamethasone.

**Figure 2:**
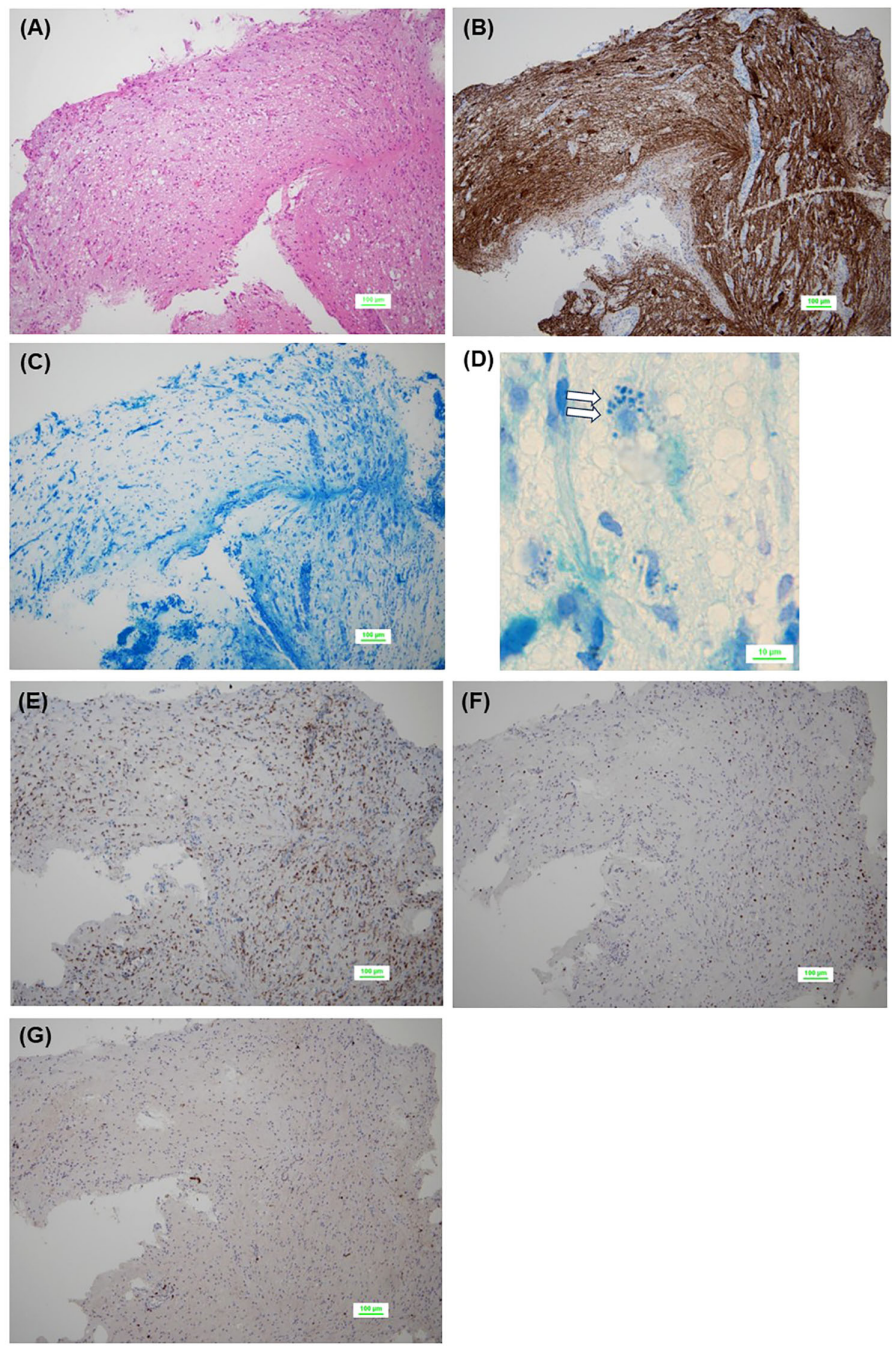
Pathological findings of initial biopsy. No tumor cells are observed in the background of the brain tissue [**(A)**, HE staining]. Residual neuroaxons are observed [**(B)**, neurofilament protein staining], but few or no myelinated structures are observed, suggesting demyelination [**(C)**, Kluver-Barrera staining]. Phagocytosis of the myelin sheath is seen (blank arrows) [**(D)**, Kluver-Barrera staining]. Abundant infiltration of CD68^+^ cells **(E)** and CD3^+^ T lymphocytes **(F)** is observed, while CD20^+^ B lymphocytes are scarce **(G)**. CD, cluster of differentiation; HE, hematoxylin-eosin.

Histologically, neurofilament protein staining showed residual axons, but demyelination and phagocytosis of the myelin sheath were observed by Kluver-Barrera staining, set against a background of brain tissue without characteristic cellular appearance (**Figures 2A–D**). Immunohistochemistry revealed abundant infiltration of CD3- or CD68-positive cells, but a paucity of CD20- or CD138-positive cells (**Figures 2E–G**). Other immunohistochemical findings were as follows: GFAP (+), S100 (+), synaptophysin (+), NF70 (+), olig2 (focal +), IDH1 R132H (−), ATRX (+, retained), and p53 (−). Pathological findings were negative for Langerhans cell histiocytosis, lymphocytic hypophysitis, neoplastic disorders, ischemia, and vasculitis, and the pathological diagnosis of the first biopsy was limited to a brain finding of suspected demyelination.

## 3 Diagnostic assessment and outcome

Although the patient was under steroid administration, we consulted neurologists and performed additional tests to differentiate the disease. Blood test findings, including anti-myelin oligodendrocyte glycoprotein, anti-aquaporin-4 (AQP4), human T-cell lymphotropic virus type 1, anti-SS, and antineutrophilic cytoplasmic antibodies, were unremarkable. The cerebrospinal fluid (CSF) cell count and protein levels were mildly elevated, with 503 cells (343 polynuclear cells) and 96 mg/dL, respectively. The oligoclonal band test was negative. Interleukin (IL)-6 levels in the CSF were markedly high at 20,200 pg/mL (reference value: less than 4), suggesting the presence of severe inflammation; however, a definitive diagnosis could not be made.

**Figure 3:**
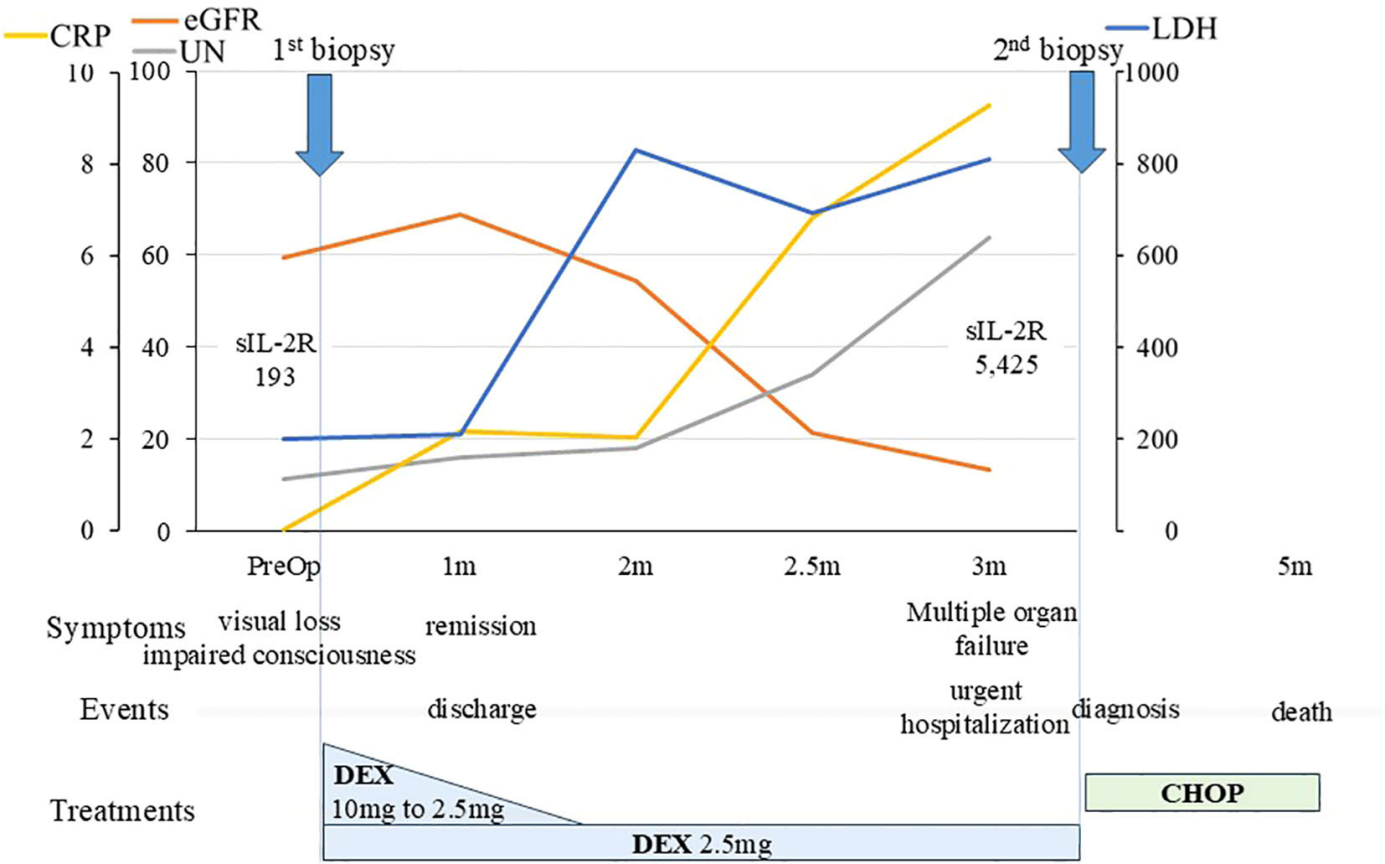
Clinical course of the patient. An overview of the patient’s progress and test results is shown. We initiated steroid treatment immediately after the initial biopsy, and a favorable response was obtained, allowing the patient to be discharged home one month after the biopsy. The patient’s general condition was maintained on 2.5 mg/day of DEX, but three months later, her condition deteriorated, necessitating emergency hospitalization. We performed a second biopsy, leading to a definitive diagnosis of lymphoma. CHOP therapy was started promptly, but unfortunately, it was ineffective. The patient ultimately passed away five months after the initial biopsy. CHOP, (cyclophosphamide, hydroxydaunorubicin, oncovin, and prednisolone); CRP, C-reactive protein; DEX, dexamethasone; eGFR, estimated glomerular filtration rate; LDH, lactate dehydrogenase; m, month; PreOp, preoperative; sIL-2R, soluble interleukin-2 receptor; UN, urea nitrogen.

**Figure 4:**
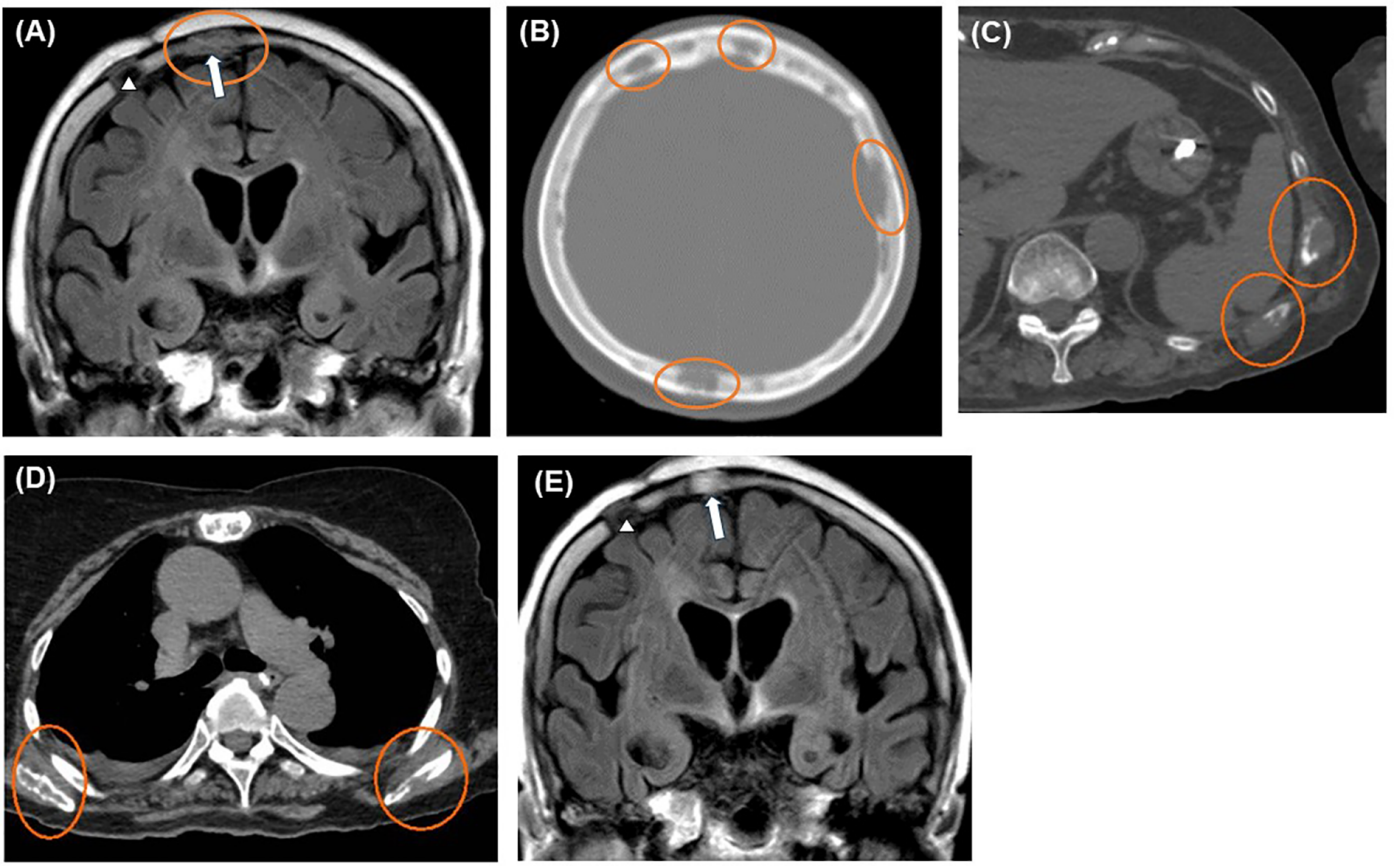
Imaging findings after exacerbation. Head MRI and CT scans revealing multiple skull lesions (orange circles) on FLAIR coronal **(A)** and CT axial sections **(B)**. Whole-body CT axial sections showing multiple bone lesions (orange circles) on the ribs **(C)** and bilateral scapulae **(D)**. The last MRI, taken four months after the initial biopsy, shows no recurrence of brain lesions **(E)**. Blank arrowheads and blank arrows indicate the scars of the initial and second biopsy procedures, respectively **(A, E)**. CT, computed tomography; FLAIR, fluid-attenuated inversion recovery; MRI, magnetic resonance imaging.

The patient continued to take 2.5 mg/day of dexamethasone. However, 3 months later, her general condition deteriorated rapidly, with multiple osteolytic changes in bones throughout the body, including the skull, hypercalcemia, renal impairment, low platelet count, and anemia (**Figures 3**, **4A–D**). Serum sIL-2R levels increased from 193 to 5,425 ng/mL; however, the brain lesions remained stable without recurrence. We performed a second biopsy of the skull, suspecting multiple myeloma. The bone marrow appeared fragile and degenerated by the lesion and could be easily curetted away. Histologically, a diffuse infiltrate of large atypical cells with enlarged nuclei and increased chromatin was observed, which were positive for CD20, BCL6, and MUM1, and negative for CD3, CD10, and CD138. The Ki-67 index was greater than 70%, and a bone marrow biopsy performed on the iliac bone showed similar findings of cellular infiltration. Bence Jones protein or M-proteins were not detected by immunoelectrophoresis. The final pathological diagnosis of the bone and skull lesions was DLBCL, non-germinal center B-cell like type (GCB). Consequently, we diagnosed the brain lesion as a sentinel lesion at this time.

We initiated combination chemotherapy with cyclophosphamide, hydroxydaunorubicin, oncovin, and prednisone (CHOP regimen), but the patient passed away five months after the initial biopsy. No recurrence of the brain lesions was observed until the end of her life (**Figure 4E**).

## 4 Discussion

This is the world’s first case of a cerebral sentinel lesion preceding systemic DLBCL arising “outside” the CNS. To summarize the patient’s course, an older woman presented with rapidly progressive visual field disturbance, and a head MRI showed an almost homogeneous contrast-enhancing lesion from the pituitary stalk to the hypothalamus. We suspected lymphoma and performed a biopsy of the brain lesion “before” administering steroids, which revealed inflammatory demyelination with T-cell dominant infiltration. The patient responded well to steroids and was discharged home. However, several months later, her condition worsened with osteolytic lesions throughout the body and multiple organ failure. A second biopsy of the skull lesion led to a definitive diagnosis of systemic DLBCL with cerebral sentinel lesion. We initiated treatment with the CHOP regimen immediately, but the patient died five months after the first biopsy. No recurrence of the brain lesions was observed from the start of the steroid therapy until death.

Preoperative differential diagnoses included PCNSL, high-grade glioma, Langerhans cell histiocytosis, and lymphocytic hypophysitis. However, the pathology from the first biopsy was negative for all of these and was only suggestive of inflammatory demyelination with T cell infiltration. Regarding demyelination, the patient’s course of rapidly progressive disease and subacute cognitive decline were atypical clinical presentations for multiple sclerosis (MS) (18, 19), and clinical findings did not satisfy the McDonald criteria, ruling out MS (20). Instead, neuromyelitis optica spectrum disorder (NMOSD) should have been considered as a differential diagnosis because circumventricular organs (CVOs), such as the area postrema or paraventricular hypothalamic nucleus, are the sites most likely to be affected by diseases caused by serum autoantibodies (including NMOSD) due to the lack of the blood-brain barrier (21, 22). We examined the anti-AQP4 antibody using the Live Cell-based assay, the most sensitive method available (23); however, the test results were negative, which led to the exclusion of NMOSD in this case.

We reviewed previous reports on PCNSL-associated sentinel lesions and identified 22 cases after excluding uncertain ones (3–17). The mean age of the patients was 58.4 (range: 36–79) years, and 72.7% (16 of 22 cases) of those with sentinel lesions were female, despite PCNSL being more common in males (24). The imaging findings of sentinel lesions are similar to those of PCNSL, including a uniform contrast effect; however, diffusion restriction tends to be relatively unremarkable (12, 13). Of the 16 cases with well-described pathology, 14 (87.5%) showed inflammatory demyelination with predominant T-cell and macrophage infiltration. Sentinel lesions generally show an excellent response to steroids, and the median time from diagnosis of the sentinel lesion to definitive lymphoma diagnosis was 6.0 (range: 3–66) months in the 19 cases described in the article. The clinical course of the present case was consistent with all of these findings.

Although the underlying mechanism of sentinel lesion formation remains unclear, several hypotheses have been proposed, with some involving immunological mechanisms. The first possibility is that we are observing a lymphoma lesion temporarily obscured by steroids (3, 9, 11, 12, 14). However, this does not apply in the present case, as steroids were not used prior to the biopsy. The next is the consequence of sampling error (9, 15). To minimize sampling errors, we performed the first biopsy after removing the ependymal layer of the third ventricle floor and collected more than ten specimens; the enhancement defect (i.e., biopsy site) was confirmed on MRI after surgery. Still, the possibility of failed sampling of the lesion cannot be ruled out, and in particular, there is a possibility that the tumor margin was sampled in this case. However, unlike steroid-treated cases before biopsy, steroid-naive cases do not show pathological demyelination in either the core or periphery of the tumor mass (25). Therefore, based on the fact that tissue showing inflammatory demyelination was collected, tissue sampling was considered to be performed correctly in this case. The third hypothesis is that some inflammatory responses to precancerous lesions of the lymphoma may later lead to lymphoma development (7, 14, 17). NF-kappa B signaling, which is also involved in inflammation, is activated in DLBCL (26). The present case showed high inflammation, with abnormally high levels of IL-6 in the CSF. However, a precancerous lesion would have caused cancer in the same site, but in this case, the lymphoma lesion developed outside the CNS, which was inconsistent with this hypothesis. The fourth hypothesis posits that nonneoplastic inflammatory cells represent immune responses against clinically occult lymphoma, which only manifests when a subclone capable of evading the immune system develops (3, 7, 10, 11, 13, 14). However, this theory would not explain why the sentinel lesions occurred in the brain, whereas the lymphoma did not occur in the brain but in the bone and skull. Additionally, we support the idea that sentinel lesions and lymphoma masses are different. Previous reports on multiple brain lesions have indicated that sentinel lesions differ from lymphoma masses and do not contain tumor cells. For example, in one case, the first biopsy showed inflammatory demyelination (=sentinel lesion), while a biopsy from another site confirmed the diagnosis of lymphoma (13). In another case, an autopsy showed the simultaneous presence of lymphoma and sentinel lesions without lymphoma cells in different areas (10).

The fifth and plausible theory is that some autoantibodies against the brain cause neuroinflammation, including the concept of paraneoplastic neurological syndrome (PNS), leading to sentinel lesions. Of the 22 prior cases with sentinel lesions, only one (4.5%) exhibited a lesion in the CVO (3), indicating that the CVO is not a common location for PCNSL-associated sentinel lesions. However, in the present case, the lesion occurred in the CVO, a site vulnerable to disorders associated with “serum” autoantibodies. The absence of oligoclonal bands indicated that the production of autoantibodies in the “CNS” was negative, suggesting that serum autoantibodies may play a role in disease pathogenesis. Autoantibodies can be produced by DLBCL cells (9, 11), but we consider this unlikely, as it does not explain why sentinel lesions preceded the early stage of the disease when the lymphoma volume was relatively low, and it is unclear whether lymphoma cells have the ability to produce effective antibodies in the first place. Thus, we assumed that autoantibodies were elicited as part of the immune response to lymphoma cells (9, 11, 13, 16). Although we could not perform sufficient testing for serum autoantibodies due to insurance limitations, the inability to detect autoantibodies does not rule out PNS (27, 28). Previous reports have documented cases of PNS associated with bone DLBCL (29, 30) and a tumor-like lesion in the hypothalamus caused by small cell lung carcinoma-related PNS (31). Based on these findings, we speculate that extra-CNS DLBCL induces serum autoantibodies, leading to hypothalamic sentinel lesions. In lesions showing significant T-cell inflammatory demyelination that do not lead to a definitive diagnosis, careful follow-up is necessary, including both systemic evaluation and monitoring of brain lesions, with the possibility of sentinel lesions in mind.

Regrettably, throughout the course, we could not perform MRIs of the spinal cord or fluorodeoxyglucose (FDG)-positron emission tomography (PET). Sentinel lesions may occur in the early stages of lymphoma formation, and small systemic lesions may have been detected if FDG-PET had been performed before starting steroids. However, the rapid progression of the disease and the need for urgent diagnostic and therapeutic interventions did not allow for a PET scan. In addition, the possibility of two independent rare diseases—bone lymphoma and autoimmune hypothalamic-hypophysitis—coinciding cannot be ruled out. If this is the case, the importance of considering close follow-up to detect potential subsequent disease remains critical. Because head MRI alone may delay the detection of lesions, the threshold for conducting a PET scan for a systemic search should be lowered. Additionally, blood tests should be performed to check for damage to the other organs should be actively considered. This approach could facilitate timely diagnosis and intervention, potentially improving outcomes for patients with early-stage lymphoma.

In conclusion, we encountered the first case of a hypothalamic demyelinating lesion preceding extracephalic DLBCL. Sentinel demyelinating lesions can occur before DLBCL other than those in the CNS. When encountering tumefactive inflammatory demyelination with T-lymphocyte-dominated infiltration in the brain, clinicians must consider the possibility of a delayed presentation of DLBCL and follow patients with caution.

## Data Availability

The original contributions presented in the study are included in the article/supplementary material. Further inquiries can be directed to the corresponding author/s.
